# Long-term usage, condition, and cultural acceptability of the BALatrine project: findings from a subsidised sanitation improvement intervention in rural Central Java, Indonesia

**DOI:** 10.1186/s40249-026-01499-6

**Published:** 2026-09-01

**Authors:** Callum Lowe, Matthew Kelly, Salvador Amaral, Novia Handayani, Bagoes Widjanarko, Budiyono Budiyono, Nissa Kusariana, Budi Laksono, Juniawati Juniawati, Muharsono Muharsono, Sukoto Sukoto, Suharyo Hadisaputro, Kinley Wangdi, Aparna Lal, Johanna Kurscheid, Susana Vaz Nery, Ricardo J. Soares-Magalhaes, Kate Halton, James McCarthy, Archie C. A. Clements, Donald Stewart, Darren Gray

**Affiliations:** 1 National Centre for Epidemiology and Population Health, Australian National University, 62 Mills Road, Acton, ACT 2601 Australia; 2 Menzies School of Health Research, Darwin, NT Australia; 3 Faculty of Public Health, Universitas Diponegoro, Semarang, Central Java Indonesia; 4 Yayasan Wahana Bakti Sejahtera, Semarang, Central Java Indonesia; 5 Politeknik Kesehatan, Kementerian Kesehatan Semarang, Semarang, Indonesia; 6 HEAL Global Research Centre, Health Research Institute, Faculty of Health, University of Canberra, Canberra, Australia; 7 Swiss TPH, University of Basel, Basel, Switzerland; 8 Kirby Institute, University of New South Wales, Kensington, Australia; 9 School of Veterinary Science, University of Queensland, Gatton, Australia; 10 Children Health and Environment Program, UQ Children’s Health Research Centre, The University of Queensland, South Brisbane, Australia; 11 Queensland University of Technology, Brisbane, Australia; 12 Walter and Eliza Hall Institute of Medical Research, Melbourne, Australia; 13 Department of Infectious Diseases, The University of Melbourne, Melbourne, Australia; 14 Queens University, Belfast, UK; 15 School of Medicine and Dentistry, Griffith University, Brisbane, QLD Australia; 16 Population Health Program, QIMR Berghofer Medical Research Institute, Brisbane, QLD Australia

**Keywords:** Water, Sanitation and hygiene, Cultural acceptability, BALatrine, Sanitation, Hygiene, Qualitative, Soil-transmitted helminth

## Abstract

**Background:**

Much work remains to achieve the sustainable development goals for improved sanitation access for all. Subsidised sanitation interventions are often criticised due to the possibility of the infrastructure being neglected or culturally inappropriate for target communities. The BALatrine intervention is a novel approach consisting of a community-wide subsidised recipient-aided installation of an improved latrine with septic tank (‘BALatrine’) and education package. This study aimed to investigate the long-term usage and cultural acceptability of the BALatrine project four to six years following its implementation in a main trial.

**Methods:**

We conducted a cross-sectional follow-up study in September 2022 of 88 households who received BALatrines in two villages between 2016 and 2017. Quantitative and qualitative interviews with the household head and members were conducted to understand BALatrine usage and perceptions. Descriptive statistics were used to tabulate interview responses.

**Results:**

The BALatrines were still in excellent condition with 89% still functional as initially intended corresponding to 95% of participants regularly passing a bowel motion in the BALatrine. A 77% reduction in self-reported open defecation was achieved. Participants expressed concerns towards the prospect of having to empty the septic tanks. Interviews with key village stakeholders demonstrated participants desire for aid and financial difficulties in obtaining and maintaining such improved sanitation.

**Conclusions:**

Follow-up results of the BALatrine intervention demonstrate that the BALatrine’s in the two surveyed villages are overall culturally acceptable and well-maintained. The BALatrine intervention may be suitable for use in other rural areas of Indonesia and the region for improving sanitation coverage.

**Supplementary Information:**

The online version contains supplementary material available at https://doi.org/10.1186/s40249-026-01499-6.

## Background

Safe water, sanitation, and hygiene (WASH) is a critical component of global health development, reflected in the United Nations 6th Sustainable Development Goal *“ensure availability and sustainable management of water and sanitation for all”* [1]. The importance of WASH is reflected by the estimated, 1.4 million deaths which could have been prevented by safe WASH in 2019, the lack of which is also responsible for 69% of diarrhoea cases [2]. These deaths and morbidity occur predominantly in low- and middle-income countries where WASH coverage is sub-optimal [3]. Efforts to improve WASH coverage globally have spanned decades. Monitoring of long-term sustainability of infrastructure and behaviour change is important to ensure that communities maintain long-term adherence and do not revert to open defecation (OD). This has proven a major challenge in many parts of the world and is a motivator to develop culturally acceptable sustainable WASH interventions in contexts where open defecation is normal and widely practiced [4–7]. This is reflected towards investigating the sustainability of WASH interventions through the lens of the recipient, considering the critical requirement to address the cultural acceptability aspect of sustainable WASH, although few long-term usage studies have been published. [8, 9]. Consistent, long-term usage of WASH interventions is critical as results appear not only in the short term (e.g. reduction in diarrhoea and parasitic infections) but also in the long-term [10]. Furthermore, community-based WASH interventions need to achieve high coverage as analysis suggest reductions in diarrhoea only occur once faecal contamination of the environment is drastically reduced beyond a minimum threshold [11].

Indonesia, an archipelago country, struggles with a large proportion of the population particularly in rural areas lacking access to improved WASH, despite rapid urbanisation in the recent decades [12]. Nearly all tap water is untreated in Indonesia, resulting in over 70% of household water samples testing positive for *Escherichia coli*, a definitive indicator of faecal contamination [13] that can arise from contamination from unimproved sanitation facilities. In 2018, 20% of the population, or approximately 53.4 million people, in Indonesia did not have access to at least basic sanitation services [14]. The tropical climate often leads to flooding in the wet season, resulting in flooded and subsequently unusable septic tanks, which causes communities to revert to OD [15]. On the other hand, water scarcity is experienced in Indonesia’s dry season, resulting in insufficient clean water. Challenges have been faced in encouraging communities to adopt improved sanitation globally and in Indonesia. Community-led total sanitation (CLTS), a widespread global strategy developed in Bangladesh which aims to encourage communities to build toilets [16] has been adopted as the primary strategy in Indonesia [17]. It is argued that supply-led or subsidized programs often lead to reversion to OD as they may not always achieve ownership and cultural acceptability [18], ‘demand driven’ approaches like CLTS provide a contrast in their attempt to stimulate ownership by recipients [19, 20]. Overall, the evidence-base is mixed.

Whilst CLTS has been valued highly in Indonesia and in many other contexts [21], controlled trials have shown CLTS fails to achieve desired increases in toilet construction in Indonesia [22], and expert observers of CLTS implementation in east Java, a part of Indonesia known for high rates of OD, noted the colonialist approach of programs using coercion, punishment and shame [23]. Experts elsewhere have critiqued CLTS for its potential human rights impacts [24]. Research in Indonesia has shown the importance of incorporating cultural norms and attitudes towards sanitation in program design [25, 26].

Considering the challenges associated with both the ‘supply led’ and ‘demand driven’ approaches, a unique approach, the ‘BALatrine’ project, was developed, whereby improved latrines are subsidised and provided to communities who, where possible, assist in their construction after attending an educational meeting. The effectiveness of the BALatrine intervention in reducing soil-transmitted helminth infections was investigated in a cluster randomised-controlled trial conducted in eight villages of rural Wonosobo, Central Java. Baseline data collection was conducted in 2016 with follow-up nine months later in 2017. The intervention consisted of the installation of a low-cost, locally designed and constructed all-weather latrine (the ‘BALatrine’) alongside community education meetings promoting good hygiene behaviours and maintenance of the BALatrine. Households that did not have sanitation facilities received the BALatrine toilet and septic tank, whereas households that already had an unimproved toilet (e.g. piped to a fishpond) were provided with a BALatrine septic tank. Further technical details surrounding the BALatrine have been described elsewhere [27, 28]. The BALatrine intervention led to a 70% reduction in infections [27].

As described, the health benefits of WASH interventions cannot be sustained if long-term use and cultural acceptability is not attained. The BALatrine project represents as a unique opportunity to investigate the compliance with and long-term cultural acceptability of a sanitation improvement intervention. A distinctive ambition of the BALatrine project was to answer the question of whether a subsidised sanitation improvement intervention can achieve high uptake and cultural acceptability by encouraging households to be part of the construction process. Long-term multi-year follow-ups of community-based sanitation improvement interventions are lacking. Therefore, in this study, we conducted a follow-up cross-sectional study of the BALatrine project to address two aims: (1) Measure the usage/compliance and condition of the BALatrines among its initial recipients and (2) Investigate the cultural acceptability of the BALatrine project.

## Methods

### Study design

**Fig. 1:**
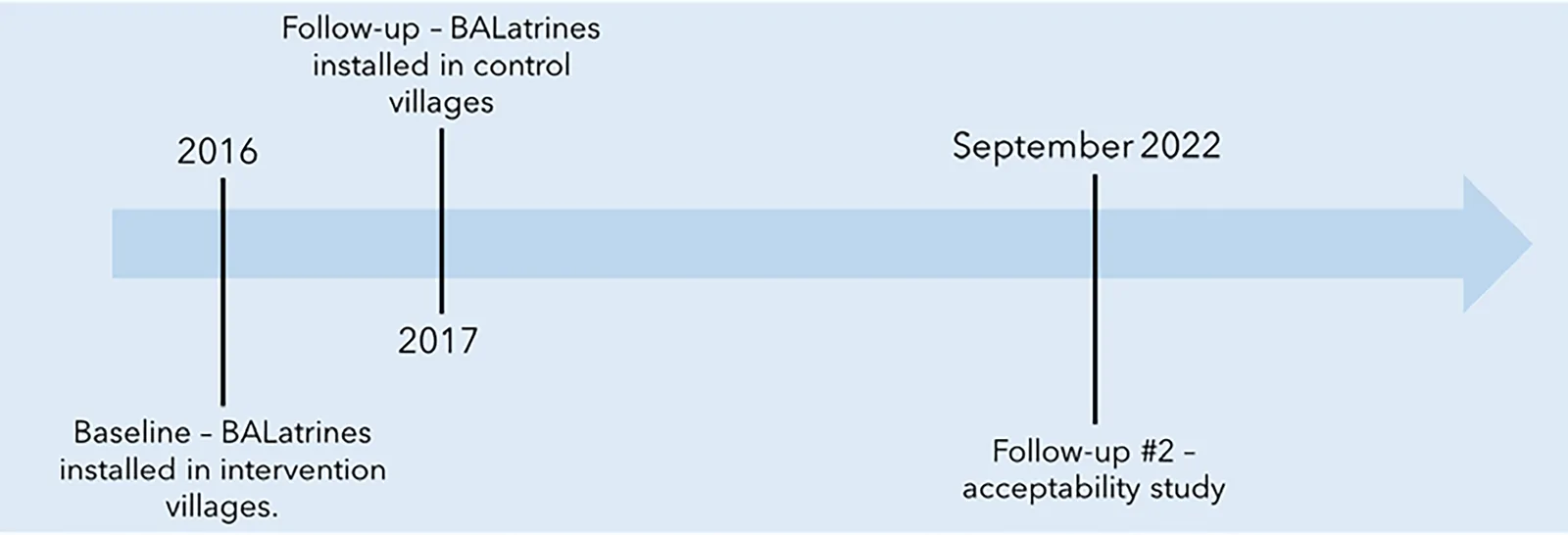
BALatrine intervention and acceptability follow-up timeline

**Fig. 2:**
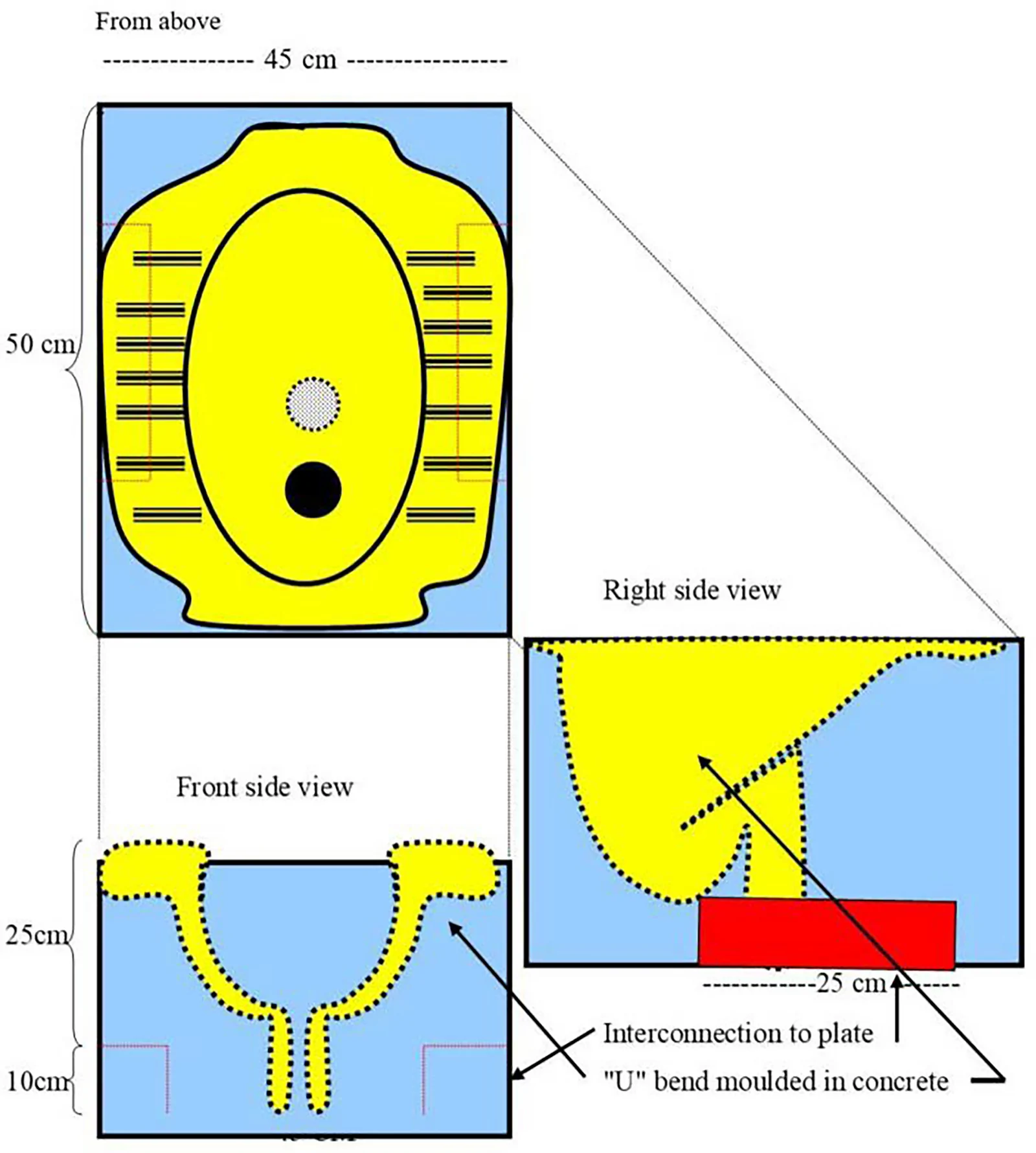
Schematic of the BALatrine toilet

This study was a mixed-methods long-term follow-up survey conducted in September 2022 (4–6 years after latrine installation - see Fig. 1), incorporating quantitative and qualitative components in villages that previously participated in the BALatrine intervention [Australian and New Zealand Clinical Trials Registry (ACTRN12613000523707)]. The villages are located in the rural Wonosobo district, Central Java province, Indonesia. This is a mountainous area with high rainfall during the wet season, typical of its tropical climate. Open defecation is practiced by approximately one third of the people living in this area, and it has one of the lowest rates of improved sanitation in Central Java (author observation). The prevalence of soil-transmitted helminth infections in these villages prior to the BALatrine intervention was approximately 15% at baseline [27]. The follow-up survey was conducted in two villages: one randomly selected control and one randomly selected intervention village from the four control and four intervention villages in the overarching BALatrine study. The control villages had BALatrines installed following the approximately 9-month trial length, following the same procedures as those in the intervention villages, with no implication on the acceptability study except for the slightly shorter follow-up length. A retrospective study design was chosen so that the condition and long-term use of the BALatrines could be measured. A long follow-up of 4–6 years was chosen to measure the long-term condition of the latrines and investigate ‘slippage’ or reversion to open defecation and other unimproved sanitation practices. A schematic diagram of the BALatrine is provided in Fig. 2.

### Sample size

The primary hypothesis was that 80% of participants would regularly use the BALatrine at follow-up based on a conservative estimate. Assuming a 5% margin of error and 80% power, a sample size of 246 persons was required. $n= \frac{{Z}^{2}\times p\times \left(1-p\right)}{{ME}^{2}}= \frac{{1.96}^{2}\times 0.8\times \left(1-0.8\right)}{{0.05}^{2}}\approx 246$. Assuming 3–4 persons would be available per household for interview; 80 households were deemed sufficient to reach the target sample size. This target was then split into 40 from a control village and 40 from an intervention village. A roster of households in the two villages from the prior intervention was conplied and then randomly sorted to obtain a set of target households.

### Study participants

Participants were individuals living in houses that had received a BALatrine as part of the BALatrine intervention trial. All individuals aged five years or older living in the household were eligible for data collection. Households were not included if there were no adults present who were permanently living in the household at the time of the BALatrine intervention trial as children may not be able to recall or speak to the technical aspects of the BALatrine. Parents aided in obtaining responses to interview questions from young children, assisting with recall for questions pertaining to retrospective sanitation behaviours.

### Definition of acceptability

It has been posited that an acceptability framework should be used in studies that measure the acceptability of WASH interventions [29]. The IBM-WASH framework has been developed to address this [30]; however we find it lacks applicability to our study as it does not provide a definition and focuses primarily on behaviour change. The theoretical framework of acceptability (TFA) developed by Sekhon et al. provides a working definition of acceptability, formed by seven key components: affective attitude, burden, ethicality, intervention coherence, opportunity-costs, perceived effectiveness, and self-efficacy [31]. We define acceptability in our study as the achievement of TFA components. The goal of this study is not to ascertain whether the BALatrine intervention was ‘acceptable’ or not, but rather to explore elements of acceptability with a mixed-methods approach. Using a hybrid-analytic approach, we created a series of themes to be explored in quantitative and qualitative data collection and then in the final part of analysis applied them to the TFA.

### Data collection

Data was collected in a single visit to households. Quantitative data collection among BALatrine households involved the administration of two short electronic tablet assisted surveys—one ‘individual’ survey answered by all individuals living in the household that met the inclusion criteria and one ‘household’ survey answered by a household head representing the household. The household survey also included a BALatrine inspection provided consent of the household head. The individual survey captured information pertaining to individuals’ bowel motion habits before and after the BALatrine intervention, attitudes towards the BALatrine intervention, and self-perceived improvements in health. The household-level interview captured information regarding the condition of the BALatrine, socio-economic status of the household, modifications to the BALatrine and history of the BALatrine septic tank ever being emptied. The BALatrine inspection captured information on the condition of the BALatrine; considering cleanliness, odour, provision of water/soap, and functionality.

Qualitative interviews provide an additional opportunity to gain insights into the acceptability of the BALatrine through dialogue and free speech that cannot be captured in a quantitative questionnaire. Qualitative interviews were conducted with all consenting members of the household together in a room where discussion would not be audible to anyone outside the home. The qualitative interviews with households were semi-structured with a list of prompts pertaining to opinions regarding the BALatrine intervention. Participants were asked about their experience with the BALatrine, any issues they have had with the BALatrine, and maintenance tasks. They were also asked about health and environmental benefits of having the BALatrine as well as whether they felt ownership of the BALatrine. We also discussed a hypothetical question of whether participants would be willing to pay for the BALatrine were it not a subsidised project. This question was asked because it points to the acceptability of the BALatrine intervention in contrast with other approaches where villagers in this region are asked to pay, although this may not reflect actual purchasing behaviour. A rough target of 15 min was determined for interview length. Qualitative interviews were conducted until data saturation was reached. Qualitative interviews were recorded, and later transcribed and Javanese speech was translated to Bahasa Indonesia. A further translation into English was conducted for the parts of speech reported in this paper.

Where possible, additional qualitative interviews were conducted with a list of key stakeholders. This decision was made as the acceptability of the BALatrine intervention depends not only on the attitudes of the recipients, but also on the attitudes of local village governance and other key stakeholders who are necessary for implementation and were involved in the intervention. Village heads, local health clinic sanitation staff, and village nurses and midwives who held their position during the time of the BALatrine intervention were identified as candidates. The key stakeholders were contacted by mobile phone and invited to participate, and a location of their choice was chosen for conducting the qualitative interview. A different set of prompts for the semi-structured interview was created with the goal of capturing views/opinions of the BALatrine intervention from a different angle (e.g. who bears the responsibility of improved sanitation, barriers to improving sanitation coverage). These interviews were also audio-recorded and transcribed. The quantitative survey was administered by local data collectors who were trained in the survey during a workshop. Data were verified on-site by a research supervisor. Data collectors were also trained in the qualitative survey during the workshop with pilot testing of the interviewer prompts.

### Qualitative analysis

We employed an inductive, thematic approach to qualitative analysis as outlined by Braun and Clarke [32]. Transcripts were combined into a single document and read multiple times. Key pieces of speech were coded, and codes were used to generate themes. Themes were then modified and re-visited after re-reading the interviews. A final set of themes was generated and key parts of speech for each theme were reported in the paper.

### Statistical analysis

Descriptive statistics involved tabulating quantitative variables such as age, sex, income, BALatrine condition, likes/dislikes of the BALatrine by frequency and proportions of each response. Frequency and proportion of the responses to prior and present bowel motion locations were tabulated and stratified by village for descriptive comparison.

### Ethics

This project received ethical approval from the Australian National University Human Research Ethics Committee (No. 2022/386) and the ethical committee of *Poltekkes Kemenkes Semarang* (Semarang Ministry of Health Polytechnic) (No. 0477/EA/KEPK/2022). Participants signed a consent form to be involved in the study and for data collectors to visit their BALatrine. Participants were given the opportunity to refuse audio recording of the qualitative interview which was conducted in privacy within their homes. The inspection of the BALatrine was only done with the consent of the household head and in privacy. Data was anonymized and kept on the principal investigator’s computer. Identifying information was removed from transcripts presented in this publication in order to protect participants privacy. This research was conducted in accordance with the Declaration of Helsinki.

## Results

### Aspects of acceptability developed in the study

Applying the TFA to the BALatrine intervention, we came up with a set of parameters that would be indicative of long-term acceptability specifically for the BALatrine intervention (Table 1). We made the addition of including long-term usage as part of the measurement of the acceptability of the BALatrine intervention. The long-term consistent usage of the intervention may have not been part of acceptability studies of other healthcare interventions that are only experienced at a single point in time (i.e. undertaking a single surgery or receiving a single-dose vaccination).

**Table 1:** Domain, definition, and parameters used to measure the acceptability of the BALatrine intervention using the theoretical working definition as proposed by Sekhon et al. [31]

Domain	Definition	Parameters
Affective attitude	How an individual feels about the intervention	Likes/Dislikes of the BALatrine
Burden	The perceived amount of effort that is required to participate in the intervention	Time commitment Ability to build/get help to build the BALatrine Burden of maintaining/emptying the BALatrine
Ethicality	The extent to which the intervention has good fit with an individual’s value system	Preferences for passing bowel motions Comfort in using the BALatrine
Intervention coherence	The extent to which the participant understands the intervention, and how the intervention works	Understanding the importance of improved sanitation Understanding how to maintain the BALatrine
Opportunity costs	The extent to which benefits, profits or values must be given up to engage in the intervention	Preference of open defecation over toilet use Loss of convenience when using a toilet requiring cleaning/emptying
Perceived effectiveness	The extent to which the intervention is perceived as likely to achieve its purpose	Perceived effectiveness on gastrointestinal illness Perceived reduction in environmental contamination
Self-efficacy	The participants confidence that they can perform the behaviour(s) required to participate in the intervention	Maintain the BALatrine Emptying the BALatrine Handwashing consistency

### Quantitative data

#### Demographics of the study sample

A total of 88 households were included in analysis corresponding to 248 participants. The demographics of the study population are presented in Table 2. Participants were approximately evenly divided by sex and were aged between 5 and 85 years old. Monthly income was below the international poverty line in some households, with 8% of households earning less than IDR 500,000 (~ USD 32) per month. Three-quarters (76.2%) of households earnt between IDR 500,000–2,000,000 (~ USD 32–129) per month. A small subset (15.9%) earnt over IDR 2,000,000 per month.

**Table 2:** Demographic characteristics of the study population and mean household income

Characteristic	*n* (%)
Age, years	
5–19	48 (19.4%)
20–39	74 (29.8%)
40–59	92 (37.1%)
60+	34 (13.7%)
Sex	
Male	132 (53.2%)
Female	116 (46.8%)
Mean monthly household income	
< IDR 500,000	7 (8.0%)
IDR 500,000–1,000,000	18 (20.5%)
IDR 1,000,000–1,500,000	28 (31.8%)
IDR 1,500,000–2,000,000	21 (23.9%)
> IDR 2,000,000	14 (15.9%)

#### Condition of the BALatrine toilets

Of 88 BALatrines, 78 (88.6%) were functional and in use (Table 3). Eight (9.1%) were functional but flowed to a river or a fishpond as a result of damage/leaking in the septic tank or the pipe connecting the latrine to the septic tank. One BALatrine was not functional and not used entirely and one BALatrine was functional but not in use due to the family’s fear of the septic tank filling and requiring emptying. Three-quarters (73.5%) of BALatrines had soap and running water immediately available, and a quarter (24.1%) had running water only available. Two BALatrines had neither running water nor soap available. 81.3% of the BALatrines were regarded clean based of the study classification criteria (see Table 3 footer). Eleven (12.5%) of the BALatrines had some type of modification (excluding re-piping to a river or fishpond), consisting of six BALatrines that had a ceramic floor installed, two that had minor reparations performed, and three where the toilet was relocated.

**Table 3:** State of the BALatrines upon inspection

Characteristic	*n* (%)
State of the BALatrine	
Functional and in use	78 (88.6%)
Functional but flowed to river or fish pond	8 (9.1%)
Functional but not in use	1 (1.1%)
Not functional and not in use	1 (1.1%)
Condition of the BALatrine	
Clean*	67 (81.3%)
Unclean	15 (18.3%)
Facilities of the BALatrine	
Soap and running water^#^	61 (73.5%)
Running water only	20 (24.1%)
Neither	2 (2.4%)
Modifications made to the BALatrine^**^	
Given ceramic floor	6 (6.8%)
Repaired	2 (2.2%)
Toilet moved	3 (3.4%)

Not in use was defined as a household head reporting that no-one living in the household regularly used the BALatrine

*‘Clean’ was defined as the absence of dirt or dirty water on the latrine squatting surface or the floor in the immediate environment around it

^#^Soap and running water were considered present if they were available in the immediate room/location of the BALatrine, and not present if they required the user of the BALatrine to move out of the room (i.e. into a kitchen) to be obtained

**Not inclusive of modifications that led to the destruction of original function of the BALatrine (e.g. re-piped to the river/fish pond)

#### Bowel motion locations

Based off the self-reported and recall data, a high proportion (94.4%) of participants normally passed a bowel motion in the BALatrine; 86.7% and 7.7% of individuals passed a bowel motion in a functional BALatrine or an unimproved BALatrine respectively (Table 4). This led to a 77% reduction in the use of public toilets, a 67% reduction in the use of neighbour’s toilets, a 79% reduction in OD in the river/bush, and a 96% reduction in the use of old unimproved latrines (86% reduction when including an unimproved BALatrine as part of this latter category). A total of 13 individuals from 7 households regularly practiced OD after the follow-up. The most common reason was not feeling comfortable using a toilet (*n* = 6), OD being a habit since childhood (*n* = 3), other general dislikes about toilets (*n* = 2), and afraid the BALatrine would fill-up (*n* = 1). In only two cases was the reason being that the BALatrine was damaged (Supplementary Table 1).

**Table 4:** Bowel motion habits before and 4–6 years after receiving the BALatrine intervention

Location of usual bowel motion	Before intervention	At 4–6 year follow-up
BALatrine	0 (0)	215 (86.7)
Unimproved BALatrine	0 (0)	19 (7.7)
River/Bush	62 (25.0)	13 (5.2)
Old unimproved latrine	184 (74.1)	6 (2.4)
Public toilet	13 (5.2)	3 (1.2)
Neighbour’s toilet	6 (2.4)	2 (0.8)

Data expressed as *n* (%). Before-intervention data based off of self-recall. The lack of data for BALatrine and unimproved BALatrine before the intervention is because BALatrines had not been installed in the study site prior to the intervention. Totals may exceed sample size where participants reported two locations for their usual bowel motion

#### Attitudes and practices towards the BALatrine intervention

**Fig. 3:**
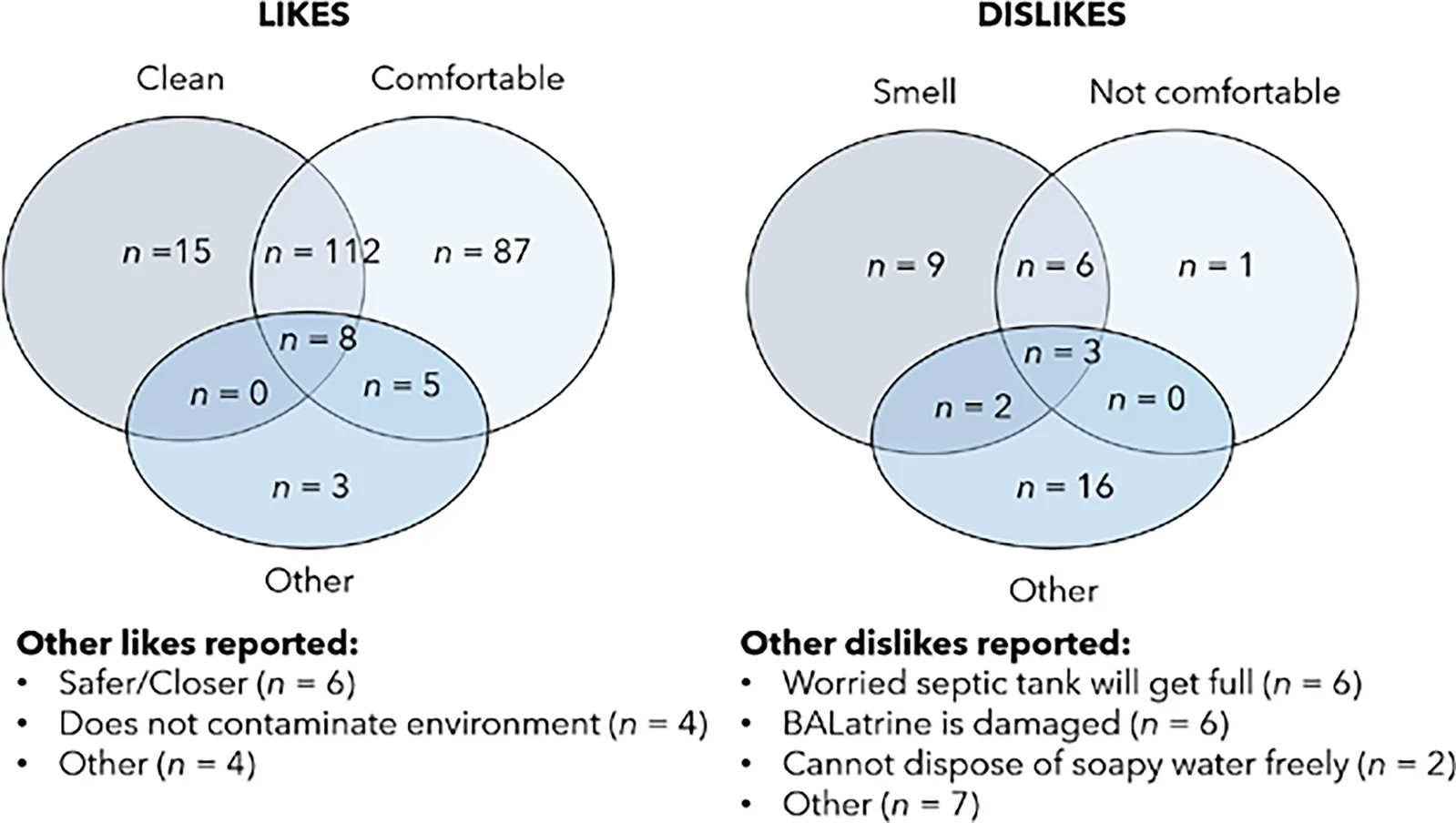
Venn-diagram of likes and dislikes of the BALatrine reported by participants. Likes/dislikes were generated inductively from participants’ responses

Generalised attitudes and practices related to the BALatrine are presented in Fig. 3 and Table 5. Nearly all (91.5%) of participants reported always washing their hands with soap after passing a bowel motion. Approximately half of participants reported experiencing less diarrhoea and worm infections since the BALatrine intervention, and a third of participants reported feeling cleaner since the intervention. When asked if participants had any particular likes or dislikes towards the BALatrine, likes were more commonly reported (Fig. 3). Approximately half of participants (54.5%) mentioned cleanliness and most (85.5%) mentioned comfortability as a positive aspect of the BALatrine. Sixteen participants mentioned other things they liked about the BALatrine. A positive health benefit was only reported by one participant. A small proportion of participants (8.1%) disliked the smell of the BALatrine, and 4.0% of participants mentioned they were not comfortable using the BALatrine. Other dislikes were reported by 21 participants, six were being worried the septic tank would get full and another six were due to the BALatrine being damaged.

**Table 5:** Attitudes and practices related to BALatrine

Characteristic	*n* (%)
Handwashing practices*	
Always	227 (91.5%)
Sometimes	21 (8.5%)
Differences in health^†^	
Less diarrhoea	139 (56.0%)
Less worm infections	120 (48.4%)
Cleaner	82 (33.1%)

*Handwashing practices following a bowel motion, self-reported

^†^Differences in health were self-perceived by participants, not prompted by interviewers, and pertained to the period after receiving the BALatrine intervention

### Qualitative data

A total of 23 recorded interviews were conducted with households and six with key stakeholders (3 interviews with village heads, 2 with local health facility sanitation staff, and 1 with a village midwife). One key stakeholder invited was unable to participate due to time constraints.

#### Accessibility of the BALatrine and comparison to prior sanitation arrangements

It emerged that participants appreciated having the BALatrine installed and participating in the intervention. Positive attitudes towards the intervention emerged including its greater accessibility compared to previous sanitation arrangements, and some participants made improvements to their BALatrine. However, not all participants felt the BALatrine was a necessity.

**Q1:** *"Yeah happy, because [we are] healthier and cleaner" - Participant*

**Q2:** *“In rainy season don't have to go outside [to pass a bowel motion], not shy, healthy, if I'm outside and get rained on I have to run.” - Participant*

**Q3:** *“Yeah…what I mean is previously [we] had to go outside, but now [we] can [go to the toilet] inside our house. So [we're] more comfortable.” - Participant*

**Q4:** *"Yeah…when we didn't have a septic tank it was so difficult...If we wanted to defecate, we defecated in the river. But now that we have a septic tank it's not difficult to defecate whenever I want." - Participant*

**Q5:*** “In the past I would be late to work as I had to defecate in the river. Now that we have a septic tank, [I can] immediately go to work.” - Participant*

***Q6:**** “Yeah...having a toilet with a septic tank is nice, it’s nicer than having one that drops right into the fishpond because the faeces aren't visible. So when looking at it it's nicer.” - Participant*

***Q7:**** “It's been given a ceramic floor, fortunately it was given to me by my boss; the leftover remains of ceramic were given to me.” - Participant*

***Q8:**** “[When asked if you had to pay, would you build a BALatrine] It seems if [I’m] not helped, I wouldn't…because there are others right, next to that house there is a public toilet.” - Participant*

#### Mixed attitudes and approaches over issues with the septic tank

Asking participants what their plans are if the septic tank gets full provides a unique insight into acceptability because it will demonstrate if a participant has the intention to take direct action to maintain the BALatrine. Attitudes of participants were mixed in this regard, with some feeling positive about the possibility, however it presented as a burden for some participants. Village leaders were also aware that the burden of emptying the septic tank may present as an issue for participants, expressing that awareness of the need to empty the septic tank must be communicated to villagers.

***Q9:**** “If its full then I will just do what I am told, if it has to be emptied that’s okay.” - Participant*

***Q10:**** “Yeah…hopefully there is help again from the government, although if for instance there wasn't then as it’s a need then we will still empty it. Still use it again.” - Participant*

***Q11****: “Yeah if there is aid for example [the BALatrine] will be renovated or made again, if someone gives us that then we would like it.” - Participant*

***Q12:**** [In response to do you feel like you 'own' the BALatrine] “Yeah I'm fulfilled with it but if it becomes full I'm not so sure.” - Participant*

**Q13:** *“At the moment I use my neighbour’s [toilet], I’m still unsure. The plan is to build [a new septic tank] but I still don’t have enough money.” - Participant*

***Q14:**** “So for toilets that get full or damaged in some way we will need some socialisation” - Village midwife*

***Q15:**** “[the septic tank] is not getting full so if it gets full [they] won't know how to deal with it, emptying it will need more socialisation.” - Village head*

***Q16:**** “There must be some socialisation to explain, because sometimes the community still thinks that it [the toilet/septic tank] doesn't really influence their health.” - Village head*

***Q17:**** “I’m happy because the water becomes clean. But lots of people who build that [septic tank] don’t use it because they are worried the water won’t flow or some other issue, lots of people are like that.” - Participant*

#### Ownership of the BALatrine

A key component of the acceptability of the BALatrine is whether participants feel a sense of ownership of the BALatrine. We consider ownership to be measured by participants’ sense of responsibility over the BALatrine, maintenance, and autonomous participation in decisions regarding the BALatrine. Varying degrees of ownership were expressed by participants and village leaders pertaining to involvement in the construction and maintenance of the BALatrine.

***Q18****: “I made it [septic tank] myself…if a mason is used at most 2 meters [depth of septic tank]…make it myself it can reach 8 meters…that was a lot of work and effort to remove all that dirt/soil” - Participant*

***Q19:**** “Yes definitely feel a sense of ownership, because they maintain it [BALatrine] and some make some additions, some are repaired.” - Village head*

#### Knowledge of improved sanitation and perceptions on intervention necessity

Some villagers expressed their awareness of the health issues associated with open defecation and unimproved sanitation. However, some participants did not understand the consequences of open defecation and local village government officials expressed doubt over whether their communities would be aware of the benefits of the BALatrine intervention.

***Q20:**** “Yeah…those bacteria or whatever it is, I heard there are lots of bacteria if we defecate in the fishpond. That's what I once heard.” - Participant*

***Q21****: “There are none [health issues], after defecating you already can't see it [the faeces]” - Participant*

***Q22:**** “But the community won't necessarily immediately become aware of…things where the benefit is not immediately seen. That's how the community thinks.” - Village head*

***Q23:**** “[after asked if participant would be willing to pay hypothetically] Yeah...maybe, previously we didn't understand, but if we understood the side effects [of unimproved sanitation] maybe we want [to build a BALatrine]. If we were asked to pay we still would want when considering the health benefits.” - Participant*

***Q24:**** “If there isn’t a [subsidy program] in this village, and if we don’t build [a BALatrine/toilet] then there will be lots of diseases, no matter what people still won’t build one.” - Participant*

#### Conflicting attitudes over who bears the responsibility to finance and repair sanitation infrastructure

It emerged that there was great conflict between the opinions of BALatrine recipients and key stakeholders regarding who bears the responsibility for finance and repair of the BALatrines. Village officials thought building and maintaining toilets was a responsibility for villagers themselves and as a result, they were of the perspective that without subsidies, it will be difficult to achieve high sanitation coverage. Some participants however felt that if one participant received a subsidised latrine, then it must be subsidised for all.

***Q25:**** “Yeah…we request to be helped, we don't [want to] pay personally. If we pay personally its better to buy [materials] at the shop if we can afford. But if we can't afford then we would request to not pay.” - Participant*

***Q26:**** “I think I wouldn't [want to build a latrine myself]. [There must be help from the] government.” - Participant*

***Q27:**** “Improved sanitation is everyone's individual responsibility. If our sanitation is good then our health will also be good.” - Village midwife.*

***Q28:**** “…they want to be helped 100% like that, that's the problem with the community…if we don't 100% pay for the building and other things it will be difficult.” - Village midwife*

***Q29:**** “Yeah…if everyone told is to pay then we must pay, but if most people don’t have to pay then we also won’t pay.” - Villager*

***Q30:**** “Yeah…if we are told to pay then no one will want to. It’s an aid program, right.” - Villager*

***Q31****: “In that village we have encouraged them to build latrines many times but they still hope for aid, because in (adjacent village) and (adjacent village) there is aid…We hoped with the stimulants in the neighbouring village that they would become triggered, but the reality is they hope for aid.” - Village midwife.*

***Q32: “****How could we get 100% [BALatrine coverage] it’s not only because of the help from the BALatrine project but because we stimulated the community with the military and others whereby they went to the village and they helped houses to the point where they wanted to open...open their door like that, if there wasn't help from government agency other than the village, military and police it would be difficult.” - Village midwife*

#### Engagement with communities for successful intervention

There was indication from village stakeholders that prior ‘jambanisasi’ (social stimulation and encouragement to adopt a latrine) was a key factor in the success of the BALatrine project. Such encouragement and social stimulation aimed to increase awareness over the need to own and use a latrine as reflected:

***Q33:**** It was different to the past, where we had to make a massive deal of jambanisasi*^*1*^*, if there wasn't any help they didn’t want it, but now praise to god they are already…already aware of the importance of building a toilet.*

***Q34****: “An indication of a healthy house is a healthy toilet, they are actually embarrassed if their house is considered not a healthy house… they are embarrassed and aware of that. And honestly if they get aid then they are happy.” - Sanitation staff*

^1^‘Jambanisasi’ is an Indonesian neologism referring to the practice of widespread community public health messaging and campaigning on the importance of installing a ‘*jamban’* (toilet).

## Discussion

Debate persists in the literature regarding whether subsidised sanitation interventions are acceptable in the long-run [33], while other approaches such as CLTS are bottom-up demand-driven in an attempt to promote self-determination in adopting sanitation infrastructure [23]. Existing follow-up acceptability studies of subsidised projects tend to have short duration, and the majority of evidence demonstrating that they fail to achieve long-term usage and behaviour change is anecdotal. We found new evidence that the subsidised approach can be successful in the long-term with the BALatrine project. The BALatrine intervention was associated with a 79% reduction in self-reported open defecation and an 86% reduction in self-reported use of unimproved latrines (latrines piped to a fishpond or river). The intervention was associated with high acceptability that was reflected in the high (89%) proportion of BALatrines that were functional and in use at follow-up corresponding to 95% of participants. The BALatrine intervention recipients displayed a sense of responsibility and a reduction in self-reported open defecation as seen in other studies in developing settings [34]. An indicator of acceptability, the condition of the BALatrines, was found to be very high with the overall majority that were fully functional and in use and deemed clean with handwashing facilities.

Ten percent of the BALatrines were modified by re-piping to a river/fishpond due to technical issues where the BALatrine septic tank was installed in soft ground prone to flooding from surrounding water bodies. The eight BALatrines that had been modified by re-routing to a fishpond or river all experienced flooding into the septic tank which is a result of the tropical climate leading to saturation of the soil. Whilst the re-routing to a fishpond or septic tank is considered unimproved and participants were educated about the environmental problems associated with this, it is likely that participants had no other choice, and wished to keep using the BALatrine due to preference of the privacy and comfort it offered participants (Fig. 3). Nevertheless, this is a limitation of the BALatrine intervention and long-term acceptability could be improved if the septic tanks were less susceptible to flooding.

A major finding from qualitative interviews indicating one of participants’ largest reasons for a positive attitude towards the BALatrine in that it provided comfort through not having to walk outside, potentially during rain, to use the toilet. These findings indicate the BALatrine can be regarded acceptable considering the ‘Affective attitude’ and ‘Ethicality’ components of the TFA (Fig. 3). The small number of BALatrines that received upgrades such as ceramic floors (Table 3) is indicative of good acceptability and self-efficacy as such upgrades were independently arranged by participants. A key component of acceptability of the BALatrine intervention is participants’ readiness and willingness to maintain the BALatrine, including taking care of the septic tank. We found this to be an area of mixed responses in qualitative interviews, with some participants reporting their preparedness to empty the septic tank if full, whereas others were worried about the financial challenges associated with this activity. There was a lack of intervention knowledge where some participants were not sure what they would do, and village stakeholders expressed doubt over whether participants will be aware of the need to empty the septic tank and that some socialisation or community engagement will be needed. This could prove to be a greater issue, and long-term usage would be lower were the follow-up period of this study to be extended. The BALatrine intervention could be improved with greater communication with participants on the expected timeframe for the BALatrine septic tanks to become full and the costs of emptying. Acceptability would likely be considerably greater if the costs of emptying septic tanks were subsidised to participants. We found that knowledge of the benefits of the BALatrine intervention was not commonly reported except for when directly asked in quantitative interviews. Rather, the accessibility was the main like reported, and health benefits were rarely discussed in qualitative interviews which was reflected in village stakeholders’ awareness of the generally low health literacy in the study area.

A review by Garn et al. identified studies measuring the impact of sanitation interventions on sanitation coverage and use but noted the scarcity of studies reporting sanitation adherence following the intervention [4]. The study found interventions on average led to a 14% increase in toilet ownership compared to controls [4]. Other studies have found that subsidised sanitation provision interventions can be successful [35], however little research has shown if subsidised sanitation provision interventions are acceptable and used in the long-term. Our findings related to the acceptability of the BALatrine intervention were similar to a subsidised sanitation provision intervention in the Gambia, where after a 25–47 month follow-up, 87.3% of latrines were usable [36] which is similar to our results finding 88.6% of BALatrines were functional and in their original condition. Similarly, in a one-year follow-up of a subsidised latrine intervention in Niger, Diallo et al. found 92.5% of adults always used the provided toilet and that 70% were deemed clean [37], which was similar to our finding of 81.3%, although the broad umbrella term ‘clean’ limits comparisons and the follow-up length is different. The study also found participants reported accessibility and privacy as the main advantage of having the provided latrine, which were also reported by many participants in our study. Interestingly, in a 1–2 year follow-up of a non-subsidised CLTS-like sanitation improvement intervention (“SSH4A, Sustainable Sanitation and Hygiene for All) conducted in 10 countries including Indonesia saw a 63% drop in sanitation coverage 1–2 years following the intervention in Ethiopia compared to 4% in Indonesia [38]. The authors pointed out that the presence of deep-water tables was associated with discontinued use of latrines in Indonesia, which is a problem also faced in a minority of households with the BALatrine. Another CLTS-like follow-up survey found a 14.5% slippage in the use of latrines 2-years post intervention [39]. This finding was from a study conducted in East Nusa Tenggara, a rural eastern part of Indonesia with different cultural and environmental conditions compared to the mountainous area of the BALatrine study site. Hulland et al. found that frequent personal contact between households and health promoters is associated with long-term coverage [40]. Following the 9-month BALatrine intervention to the time of the acceptability follow-up, we did not conduct any follow-up or compliance checking which would suggest high acceptability and sustained use in the absence of contact. However, we cannot rule out government messaging and other messaging that could influence compliance to the BALatrine, such as existing CLTS programs and ODF campaigns. These findings demonstrate the BALatrine intervention has one of the highest long-term adherence rates among subsidised sanitation interventions reported. The work of Guiteras et al. in Bangladesh demonstrated that a CLTS program had no impact on latrine adoption unless it was accompanied by a subsidy program [34]. Whilst our study design did not include a CLTS component or control, findings from qualitative interviews demonstrated participants have little desire to pay for a sanitation intervention of this kind even if the appropriate messaging surrounding the importance of improved WASH is provided. Evidence elsewhere has highlighted the importance of emphasis on proper use and maintenance of provisional sanitation infrastructure to improve long-term intervention coherence as observed in the BALatrine project [41].

Some of the BALatrines inspected were re-routed to a fishpond or a river due to technical issues with the septic tank, most frequently due to seepage into the septic tank from fishponds or due to flooding typical of this climate. High population and housing density in these small rural villages is likely the main contributor to this practice. These issues have been negatively associated with use in other studies [4]. At baseline, only a quarter of participants were truly practicing open defecation, with the majority using a type of unimproved squat toilet above a fishpond or unimproved latrine routed to a river (Table 3). These villages are therefore different to other contexts in that the practice of using a toilet of some kind was common. Thus, the BALatrine intervention may have different results if implemented in a community where using toilets is taboo.

The overall sanitation development strategy in Indonesia relies primarily on a CLTS-like approach. Over half of households in the study had monthly incomes less than USD 96 (Table 2), which would make it a heavy financial burden to purchase the BALatrines themselves which are estimated to cost an individual around USD 50 (Lowe et al., manuscript under preparation). Participants in this study reported financial insecurity as income came primarily from agriculture and livestock which is inherently a variable output. As such, purchasing latrines with loans as pushed in CLTS campaigns is ethically questionable and financially risky, particularly in settings like rural Indonesia where income required to achieve other basic health outcomes such as sufficient dietary diversity is insufficient for the majority [42]. We found that participants do have a desire for improved sanitation, and a proportion are aware of the health benefits, which would be described in a CLTS-like campaign. However, this behaviour change communication alone is not enough to trigger purchase of improved sanitation, as nearly all interviewed participants reported if were they requested to pay they would have refused, even when aware of the health benefits. Harvey et al. found that CLTS campaigns were least effective in villages where subsidised sanitation had already been implemented [43]. We suspect that this may be due to a principle of equality, where households feel they deserve a subsidised latrine due to observing neighbouring households receive one, although we did not measure this in the questionnaire. We forecast this issue would be prominent in our study sites as we found village governance to hold strong opinions that villagers would expect to not have to pay for sanitation infrastructure. An important consideration that we observed among participants is the view that if one neighbour receives a subsidised toilet, then so should everyone else. This dependency on aid has been observed elsewhere [44]. This would put pressure on subsidised projects like the BALatrine to not discriminate between households in worthiness of receiving a subsidy. Such an approach however also has the challenge whereby communities may hold the expectation that the intervention, and further aid, will be given for free and a dependency is developed. A possible solution is to build latrines community-wide, as done in the BALatrine intervention. Stemming from this however is the concern that assets provided for free will then be neglected and hold no value to their recipients [18]. Many participants also independently made modifications to the BALatrine. Where the BALatrine septic tank remains leak-free, it is expected it should not require emptying for at least ten years, which poses little financial and time pressure.

Our study is strengthened by the inclusion of a diverse set of long-term usage and acceptability measures, including more measures than the majority of other acceptability studies of WASH interventions [29]. We also employed a follow-up between 4–6 years, which was longer than other studies [29]. This is a unique feature of our study. An earlier follow-up may overestimate long-term usage of the BALatrine as use may decline over time as people revert to open defecation or other unimproved means gradually over time. By employing a long follow-up, we have provided a more realistic interpretation of true acceptability. Our study is not without some noteworthy limitations. Our sample size was small, and the study was only conducted in two out of eight villages originally part of the BALatrine trial. There may be village-level differences that affect long-term acceptability such as socio-economic status, availability of water, existence of WASH ambassadors/promoters that are not accounted for in our study. Social-desirability bias is a prominent issue in studies where participants have been provided with something of value [45]. Whilst we made reasonable attempt to ensure participants that negative responses were acceptable, we cannot rule out potential bias in this regard. Participants were also willing to show the condition of the BALatrine which indicates they unreluctantly shared their opinions. It is important to note that the intervention in some households only involved the re-piping of a toilet from a fishpond or river to a septic tank, and therefore did not require any behaviour change in defecation habits which would bias towards greater perceived effectiveness and acceptability compared to studies conducted in areas of near universal open defecation. However, adopting a septic tank still requires added work (e.g. digging, emptying), responsibility, management, and particularly in tropical countries such as Indonesia, a risk of leaking after heavy rain that can be extremely hazardous. Thus, the acceptability of the intervention was a reasonable variable for all households and participants. We can also not rule out that unmeasured variables such as sanitation awareness campaigns occurred between installation of the BALatrines and the follow-up. However, we observed no widespread WASH messaging that was not out of the ordinary during the follow-up period that would have any major impact on the long-term usage and acceptability of the BALatrines (author observation). An education component was part of the BALatrine intervention, so it would be nearly impossible to tell whether participant’s knowledge and practices were influenced by the BALatrine education package or from an external program. Lastly, the qualitative questionnaire was conducted with all consenting household members together which could lead to suppression of perceptions/views held by some household members if conflicting with that of another household member.

## Conclusions

The BALatrine intervention is a hygiene improvement project that attracts positive attitudes and ownership from its recipients. The condition of the BALatrines 4–6 years after their installation was good with the exception of a small percentage that experienced modification due to flooding/water seepage into the septic tank. There was some doubt over the long-term maintenance tasks such as emptying the septic tanks which was driven by participants’ preference to receive subsidised assistance. As such, a subsidised sanitation intervention with an education package like the BALatrine intervention is effective and suitable for scale-up in other rural areas of Indonesia and the world.

## Supplementary Information


Supplementary material 1.

## Data Availability

The datasets generated and analysed during the current study are not publicly available due to ethical considerations but are available from the corresponding author on reasonable request.

## Abbreviations

CLTS: Community-led total sanitation
IDR: Indonesian rupiah
OD: Open-defecation
ODF: Open-defecation free
TFA: Total Framework of Acceptability
USD: United states dollar
WASH: Water, sanitation and hygiene
