# Supplementary material for: Long-term usage, condition, and cultural acceptability of the BALatrine project: findings from a subsidised sanitation improvement intervention in rural Central Java, Indonesia
Source: Infect Dis Poverty. 2026 Sep 1;15:95. doi: 10.1186/s40249-026-01499-6 (PMC13532005; doi:10.1186/s40249-026-01499-6)
Supplement: Supplementary file 1 [file 40249_2026_1499_MOESM1_ESM.docx]

**Supplementary Material**

|  | **N (%)** |
| --- | --- |
| More comfortable | 6 (2.4%) |
| Dislike toilets | 1 (0.4%) |
| Habit since childhood | 3 (1.2%) |
| BALatrine is damaged | 2 (0.8%) |
| Feel disgusting using toilet | 1 (0.4%) |
| Afraid BALatrine will fill up | 1 (0.4%) |

**Supplementary Table 1.** Reasons for open defecation following the BALatrine intervention.

|  | **Village 1 (n=120)** | **Village 2 (n=142)** | **p-value** |
| --- | --- | --- | --- |
| **Pre-intervention** |  |  |  |
| Public toilet | 9 (7.5%) | 4 (3.1%) | 0.11 |
| Neighbour’s toilet | 1 (1.0%) | 5 (4.0%) | 0.13 |
| River/Bush (OD) | 44 (37.0%) | 18 (14.0%) | <0.0001 |
| Unimproved toilet | 66 (54.5%) | 115 (30.0%) | <0.0001 |
| **4-6 years post-intervention** |  |  |  |
| BALatrine | 120 (100.0%) | 114 (89.1%) | <0.001 |
| Public toilet | 0 (0) | 3 (2.3%) | 0.10 |
| Neighbour’s toilet | 1 (1.0%) | 1 (1.0%) | 1 |
| River/Bush (OD) | 0 (0) | 13 (10.2%) | <0.001 |
| Unimproved toilet | 0 (0) | 6 (4.7%) | <0.05 |

**Supplementary Table 2**. Normal location of bowel motion pre- and post-intervention stratified by village. P-value obtained from chi-square test of proportions. OD, open defecation.
